# Neuromuscular fatigue induces phase-specific multisegmental kinematic adaptations in elite traditional rowers: a three-dimensional analysis

**DOI:** 10.3389/fspor.2026.1950960

**Published:** 2026-10-05

**Authors:** Alfonso Penichet-Tomas, Patxi León-Guereño, Arkaitz Castañeda-Babarro

**Affiliations:** 1 Health, Physical Activity and Sports Technology (Health-Tech), Physical Education and Sports, Faculty of Education, University of Alicante, Alicante, Spain; 2 Health, Physical Activity and Sports Science Laboratory, Department of Physical Activity and Sports, Faculty of Education and Sport, University of Deusto, Bilbao, Spain

**Keywords:** catch phase, center of gravity, finish phase, fixed-seat rowing, optical motion capture, postural control, rowing technique

## Abstract

**Introduction:**

Neuromuscular fatigue disrupts rowing technique, yet its multisegmental effects in Traditional fixed-seat rowing remain poorly understood. This study examined the phase-specific impact of fatigue on joint angles, body segment heights, and center of gravity position at the catch and finish phases.

**Methods:**

Nineteen elite male Traditional rowers (age: 29.4 ± 7.0 years, height: 182.0 ± 6.1 cm, body mass: 78.2 ± 8.0 kg) competing in the ACT, the highest Traditional rowing league division, were recruited for a within-subject pre–post design; two did not complete the post-fatigue assessment and marker availability varied, so paired analyses included ≤17 complete cases per variable. Following a pre-fatigue kinematic assessment, participants performed a standardized incremental test to volitional exhaustion on a fixed-seat ergometer. Three-dimensional kinematic data were captured using an STT Systems 3DMA. Paired-samples *t*-tests compared pre- and post-fatigue conditions, with Benjamini–Hochberg correction for multiple comparisons and Cohen's *d* as effect size.

**Results:**

Body segment heights decreased significantly after fatigue and remained significant after correction. At the catch, head (1,182.0 vs. 1,163.8 mm), shoulder (914.3 vs. 882.9 mm) and hand heights (641.1 vs. 626.9 mm) were reduced (adjusted *p* ≤ 0.042; *d* = −0.72 to −1.19); at the finish, head (1,073.7 vs. 1,049.0 mm) and hand heights (989.3 vs. 962.8 mm) were reduced (adjusted *p* = 0.024; *d* = −0.78 and −0.79). The vertical center of gravity decreased at both the catch (659.4 vs. 647.9 mm; adjusted *p* = 0.045; *d* = −0.64) and finish (565.2 vs. 551.5 mm; adjusted *p* = 0.045; *d* = −0.64). Changes in joint angulations (increased shoulder and elbow flexion, decreased knee flexion) showed unadjusted trends but did not survive correction and are reported as exploratory.

**Conclusion:**

Neuromuscular fatigue induces distinct, phase-specific kinematic adaptations in Traditional rowing, characterized by a systematic reduction in postural height (all three upper-body landmarks at the catch, and head and hand heights at the finish) and exploratory joint-angle adjustments at the catch and finish. These findings support multisegmental three-dimensional kinematic analysis as a sensitive tool for monitoring fatigue-related technique changes in Traditional rowing, with implications for training design and potential relevance for injury prevention.

## 1 Introduction

The seating arrangement in rowing determines not only how the rower interacts with the boat, but also the entire pattern of muscular coordination required to propel it (1). Olympic rowing relies on a mobile seat that travels along fixed rails, allowing the lower limbs to extend fully and drives the boat forward during each stroke (2). Traditional rowing, by contrast, operates with a fixed seat: the rower's pelvis remains anchored throughout the effort, fundamentally altering how propulsive force is produced and distributed across body segments (3). Whereas in Olympic rowing the legs generate approximately 45% of the propulsion impulse, with the trunk and arms accounting for roughly 32% and 23% respectively (4), the fixed seating position of Traditional rowing shifts this balance markedly: the lower limbs contribute approximately 40% of the effort, while the trunk and upper limbs together supply the remaining 60% (5, 6). Such redistribution modifies the mechanical and coordinative demands of the stroke, emphasizing the need for modality-specific approaches to technique assessment and training.

The efficiency of the rowing stroke depends on the precise sequencing and synchronization of body segments throughout each cycle, with small deviations in joint positioning at critical moments having measurable consequences for force transfer and propulsive output (2, 7). The catch and finish positions are considered critical technical events because they define the initiation and completion of force transmission through the kinetic chain during each rowing stroke (8). Moreover, the angular configuration of the trunk, shoulder, elbow, and knee at the catch and finish positions is among the most technically sensitive variables in this regard (3). These joint angles represent fundamental kinematic indicators of technique, as they determine the mechanical effectiveness of force application and the coordination between segments (9). In Traditional rowing, the permanent immobility of the seat imposes a distinct postural constraint that shapes these angles throughout the stroke, producing biomechanical profiles that differ substantially from those seen in Olympic rowing (6). Calavia-Carbajal et al. showed that trunk velocity predicts rowing output more reliably in Traditional rowing than in Olympic rowing, where leg speed dominates performance (10). This finding reinforces the role of the trunk as a central mechanical and coordinative element, particularly under fatigue conditions where its capacity to stabilize and transmit force may be compromised (11). This increased reliance on the trunk also implies the shoulder and elbow as primary contributors to force generation in the final stages of the drive. Li et al. demonstrated in elite Olympic rowers that the shoulder exhibits some of the largest joint displacements of the entire stroke cycle (12), and that its range of motion increases markedly at higher stroke rates. In Traditional rowing, the fact that the fixed bench structurally amplifies upper-limb demand suggests that the shoulder and elbow angles at the catch and finish positions may serve as particularly sensitive indicators of technical change under fatigue.

The trunk fulfills a structurally critical function in rowing: it must transmit the forces produced by the lower limbs to the oar handle through a series of mobile vertebral joints, requiring active muscular stabilization throughout the drive phase (2, 13, 14) and the mechanical demands generated by this task are considerable. In addition to its role in force transmission, the trunk is essential for maintaining vertical posture and segment alignment (12), both of which directly influence segment heights and the position of the center of gravity. In Traditional rowing, the absence of seat travel means that the trunk must absorb and redirect a larger share of the propulsive demand than in Olympic rowing (10). As a result, fatigue-related alterations in trunk function may manifest not only through changes in joint angles but also through reductions in segmental height and alterations in whole-body center of gravity.

Muscle fatigue is a well-established disruptor of motor performance, broadly understood as the progressive reduction in the capacity to generate or sustain voluntary force because of prior contractile activity (15). It manifests through both peripheral mechanisms, impairments at or distal to the neuromuscular junction affecting the contractile apparatus, and central mechanisms involving a reduction in the descending voluntary neural drive to the working musculature (16). In the present study, fatigue is operationally defined as the inability to maintain the prescribed power output, a criterion consistent with neuromuscular fatigue as indexed by task failure in the absence of direct neuromuscular measurement. From a biomechanical perspective, fatigue is particularly relevant because it disrupts intersegmental coordination and compromises the stability of movement patterns. In rowing, these processes unfold in a context of exceptionally high whole-body muscle mass engagement, estimated at approximately 70% of total body musculature (4). Fatigue also appears to affect spinal mechanics in planes other than the sagittal: frontal plane angular displacement at L3 increased significantly over the course of an incremental rowing test, and this change was better predicted by rising stroke rate than by blood lactate accumulation (17). These findings are clinically relevant given the high prevalence of musculoskeletal injuries in rowing, suggesting that fatigue-induced kinematic alterations may contribute to increased mechanical stress on the spine and associated injury risk (18).

The biomechanical constraints of Traditional rowing make direct extrapolation from Olympic rowing findings problematic (6). Without seat travel, the trunk bears a disproportionate share of propulsive demand, and the shoulder and elbow joints sustain greater relative loading per stroke. Under these conditions, fatigue could plausibly disrupt not only trunk mechanics but also shoulder, elbow, and knee angles, segment heights, and center of gravity position, the variables that jointly define the rower's postural configuration throughout the stroke (3). Within this context, fixed-seat adapted ergometers provide a controlled and reproducible platform for investigating the biomechanical demands of Traditional rowing and the effects of fatigue on rowing technique (3, 10). However, despite the growing participation and competitive expansion of Traditional rowing in recent years, scientific evidence regarding this modality remains scarce, particularly concerning the integrated multisegmental response to fatigue across the key phases of the rowing stroke.

Therefore, the aim of the present study was to examine the effects of fatigue on the multisegmental kinematics of Traditional rowing technique, assessing trunk inclination, shoulder flexion, elbow flexion, and knee flexion angles, head, shoulder, and hand heights, and the position of the center of gravity at both the catch and finish phases, before and after a standardized fatigue protocol performed on a fixed-seat adapted rowing ergometer. We hypothesized that fatigue would induce phase-specific kinematic adaptations, with reduced knee flexion and compensatory shoulder advancement at the catch, and increased elbow flexion at the finish as the arm pull compensates for diminished leg and trunk drive. In addition, we hypothesized a systematic reduction in segment heights and vertical center of gravity position at both phases, which we tentatively attributed to a possible reduced capacity of the trunk extensors to maintain postural height under the disproportionate mechanical loading of the fixed-seat configuration.

## 2 Materials and method

### 2.1 Experimental design

This study employed a within-subject pre-post design conducted in a single session. All testing took place in an indoor laboratory setting. Participants were instructed to abstain from high-intensity physical activity for 48 h prior to the testing session, to arrive adequately hydrated, and to replicate their habitual dietary intake in the 24 h preceding the test (5, 19).

Participants completed a standardized 10-minute submaximal rowing warm-up on the fixed-seat ergometer at a self-selected stroke rate and a perceived exertion of 5–6 on the Borg CR-10 scale (20), consistent with procedures reported in the Traditional rowing literature (3, 10). Following a 2 min seated rest, the pre-fatigue kinematic assessment was carried out, capturing segmental angles, body segment heights, and center of gravity position at the catch and finish phases of the rowing stroke.

Immediately following the pre-fatigue assessment, participants performed an incremental test to voluntary exhaustion on the same stationary ergometer. Once the test was completed, participants rested passively for 5 min (during which anatomical landmark positions were verified and any displaced markers were repositioned to their pre-marked locations), a recovery interval consistent with the post-exercise assessment protocols used in rowing research (21). Next, the post-fatigue kinematic assessment was conducted under conditions and using procedures identical to those of the pre-fatigue measurement.

### 2.2 Subjects

Nineteen elite male Traditional rowers (29.4 ± 7.0 years, 182.0 ± 6.1 cm, 78.2 ± 8.0 kg) competing in the ACT (Asociación de Clubes de Traineras), the highest division of the Traditional rowing league, volunteered to participate in this study (paired analyses on ≤17 complete cases per variable). Nineteen rowers completed the pre-fatigue assessment; two did not complete the post-fatigue condition owing to technical problems with the three-dimensional motion-capture recording (unrelated to the participants or the fatigue protocol), so the paired comparisons were performed on the participants with complete data in both conditions. Inclusion criteria were a minimum of five years of competitive rowing experience (range 5–23 years) and current active participation in the ACT league. Participants were excluded if they reported any musculoskeletal injury of the lumbar spine or a history of low back pain, any lower- or upper-limb injury within the six months prior to testing, or any medical condition that could compromise maximal exertion. All rowers followed the same supervised training programme, consisting of 2–3 h of training per day, 7 days per week, although the specific training load varied according to the competitive season. Prior to participation, each athlete received both oral and written information regarding the purpose and procedures of the study and provided written informed consent. Failure to provide written consent constituted an exclusion criterion. The sample comprised the elite fixed-seat rowers available for testing. To characterize the adequacy of this sample, a power analysis was performed using G * Power (version 3.1.9.7; Heinrich-Heine-Universität Düsseldorf, Germany) for a two-tailed paired-samples *t*-test: assuming a medium-to-large effect [*d* = 0.70; based on prior research reporting large fatigue-induced reductions in neuromuscular function in elite rowers (21)], *α* = 0.05 and powe*r* = 0.80, the corresponding required sample was 19 participants. Because the analyzed sample size varied across outcomes owing to marker availability (*n* = 13–17), a sensitivity analysis was conducted across this range: at *α* = 0.05% and 80% power (two-tailed), the minimum detectable effect ranged from *d* = 0.72 (*n* = 17) to d = 0.85 (*n* = 13). The study was conducted in accordance with the Declaration of Helsinki and was approved by the Ethics Committee of the University of Deusto (ETK-13/18–19).

### 2.3 Instrumentation

#### 2.3.1 Optical tracking system

Three-dimensional kinematic data were collected using the STT Systems 3DMA optical motion capture system (STT Systems, San Sebastián, Spain), operating with a six-camera configuration at a sampling frequency of 100 Hz. The system tracks the three-dimensional position of retroreflective markers with sub-millimeter accuracy specified by the manufacturer (STT Systems, San Sebastián, Spain); the session-specific residual calibration error was not available for retrospective reporting. Marker-based optical systems of this type are the accepted reference standard for three-dimensional kinematic analysis, with a precision of 0.5–1.5 mm and reliability (ICC > 0.90) reported for this class of systems (23); no independent validation of the specific STT 3DMA system is available, and the sub-millimetre accuracy is a manufacturer specification. The 3DMA system has been employed in peer-reviewed kinematic research (24, 25). Study-specific test–retest reliability was not computed; each reported value is the mean of 14 stroke cycles, which reduces random measurement error. The 3DMA whole-body protocol was applied, enabling simultaneous capture of all body segments throughout the complete rowing stroke cycle.

Prior to each testing session, the system was calibrated according to the manufacturer's standardized calibration wand procedure, performed identically for all participants to ensure consistency and reproducibility across trials. Retroreflective markers were attached directly to the participant's skin using hypoallergenic double-sided adhesive discs at the following anatomical landmarks: vertex of the head, C7 and T4 spinous processes, bilateral acromion processes, lateral epicondyles of the humerus, and bilateral wrist styloid processes for the upper body; L1 and S1 spinous processes and bilateral posterior superior iliac spines for the lumbopelvic region; and bilateral greater trochanters, lateral femoral epicondyles, lateral malleoli, and heel for the lower limbs (14, 26). Marker placement followed established protocols for whole-body kinematic analysis in rowing (14). Anatomical landmark positions were marked on the skin prior to the session. Markers remained attached throughout the session, and three-dimensional data were recorded only during the pre- and post-fatigue assessments; any markers displaced during the incremental test were repositioned at rest onto these pre-marked landmarks to reduce repositioning error between conditions.

Segmental joint angles were computed in the sagittal plane, following the marker-based definitions of Pollock et al. (14). Trunk inclination was defined as the angle between the greater-trochanter-to-acromion vector and the horizontal (positive values indicate a forward lean, negative values a backward lean). Shoulder flexion was defined as the angle between the upper-arm vector (acromion-to-elbow) and the trunk vector (acromion-to-greater-trochanter); elbow flexion as the angle between the forearm (elbow-to-wrist) and upper-arm (elbow-to-acromion) vectors; and knee flexion as the angle between the thigh (knee-to-greater-trochanter) and shank (knee-to-ankle) vectors. Given the symmetrical nature of fixed-seat ergometer rowing, angles were computed from the right side and used consistently across conditions. Positive values indicate flexion, whereas negative values indicate extension beyond the neutral position; accordingly, negative trunk and shoulder angles at the finish reflect the characteristic posterior lean and shoulder extension adopted at the end of the drive. Segmental heights of the head, shoulder, and hand were derived from the vertical coordinates of the vertex, acromion, and wrist markers respectively, expressed relative to the laboratory floor reference frame. The whole-body center of gravity was computed by the 3DMA software as the mass-weighted sum of the estimated segmental centers of mass. Although the specific set of body-segment inertial parameters implemented in the proprietary software is not publicly documented by the manufacturer, the 3DMA system has been used for three-dimensional kinematic analysis in peer-reviewed research (24, 25). The center-of-gravity position along the anteroposterior (X) axis is expressed relative to the origin of the laboratory coordinate system, which was defined by a calibration frame positioned in front of the ergometer; negative values therefore indicate the rower's position behind this origin, being less negative at the catch (body positioned forward, closer to the origin) and more negative at the finish (body reclined backward, farther from the origin). The catch position of each stroke was identified as the instant of maximum anterior displacement of the wrist marker along the anteroposterior axis (X), and the finish position as the instant of maximum posterior displacement of the same marker, consistent with established kinematic criteria for event detection in rowing (14).

#### 2.3.2 Rowing ergometer

All testing was conducted on a Concept2 Model D rowing ergometer (Concept2 Inc., Morrisville, VT, USA). The standard sliding seat was mechanically locked in a fixed position, preventing any anteroposterior displacement of the pelvis during the stroke. This configuration constrains the rower to a stationary seated posture throughout the entire stroke cycle, which is the defining characteristic that distinguishes Traditional rowing from Olympic disciplines (3, 27). Each participant's foot stretcher was individually adjusted so that the knees remained in a comfortable semi-flexed position at the catch, replicating the lower-limb angle adopted in competition (19). A damper setting corresponding to a drag factor of 140 was applied uniformly across all participants and sessions, as this resistance level has been shown to best approximate the hydrodynamic load experienced during fixed-seat on-water rowing (10, 19). The fixed-seat configuration was selected to replicate the biomechanical demands of Traditional rowing competition, in which the locked pelvis places a disproportionately greater demand on the trunk and upper limbs than in Olympic rowing (3, 5). Under this constraint, the lower limbs function primarily as a stabilizing base rather than as a dynamic propulsive element (27), a condition that the mechanically locked seat of the Concept2 ergometer faithfully reproduces.

### 2.4 Protocol

#### 2.4.1 Kinematic data collection

In both the pre-fatigue and post-fatigue conditions, each participant performed a continuous recording of at least one minute of rowing on the ergometer while three-dimensional kinematic data were captured. During both measurements, participants were instructed to maintain a constant cadence of 20 strokes·min^−1^ and an intensity corresponding to 70% of the estimated VO₂max power output, predetermined based on each participant's training records and known to them prior to the session. The target cadence and intensity were displayed on the ergometer monitor and supervised in real time; however, because the instantaneous stroke rate and power output of each recording were not logged, an actual intensity difference between the pre- and post-fatigue conditions cannot be excluded.

Participants did not receive real-time visual feedback on joint angles or the position of body segments during the recording, in order to avoid motor adaptation biases. Similarly, no technical instructions or corrections regarding stroke execution were provided, allowing each rower to perform the stroke according to their usual technical pattern.

For each participant, the 14 central consecutive stroke cycles were analyzed under pre- and post-fatigue conditions, obtained by excluding the first three strokes (start-up phase, before cadence stabilized) and the last three strokes (where technique may be subjectively altered as the rower anticipates the end of the recording) to ensure steady-state rowing and avoid transient effects associated with stroke initiation and cessation, consistent with procedures reported in the rowing kinematics literature (12). From these cycles, trunk inclination, shoulder flexion, elbow flexion, and knee flexion were extracted at the catch and finish positions as defined in Sec. 2.3.1. Additionally, head, shoulder, and hand heights were obtained from the vertical coordinates of their respective markers, and the position of the body's center of gravity was calculated along the anteroposterior (X) and vertical (Y) axes. For each analyzed variable, the mean value of the selected cycles was used in the statistical analysis.

#### 2.4.2 Neuromuscular fatigue protocol

Participants performed the standardized warm-up described in Sec. 2.1 prior to beginning the incremental test. Subsequently, an incremental test to volitional exhaustion was carried out on the same ergometer (drag factor 140). The protocol followed the methodology proposed by Mujika et al. (19), beginning at 150 W and increasing by 35 W every 4 min until exhaustion. Exhaustion was operationally defined as the inability to maintain the prescribed power output for five consecutive strokes, a criterion consistent with established incremental testing protocols in rowing research (28, 29). At exhaustion, participants reached a heart rate of 187.5 ± 8.9 bpm (*n* = 17), a rating of perceived exertion of 9.2 ± 0.8 (Borg CR-10; *n* = 19), and a peak power of 333.6 ± 30.3 W (*n* = 18) at a stroke rate of 40.3 ± 3.1 strokes·min^−1^ (*n* = 18); time to exhaustion, derived from the number of completed 4-min stages, was 23.1 ± 3.8 min (*n* = 18). All participants terminated the test under the prescribed task-failure criterion. These values, consistent with graded-test data reported for fixed-seat rowers (30, 31), confirm that a state of high systemic fatigue was elicited.

### 2.5 Statistical analysis

Statistical analyses were performed using Jamovi (version 2.5). Descriptive statistics for all variables are reported as mean ± standard deviation. Analyses were performed on complete cases for each variable; because individual markers could not be tracked reliably in some participants during one or both conditions, the analyzed sample size varied across variables (*n* = 13–17) and is reported for each outcome in Table 1. For each variable, the mean pre–post difference is reported together with its 95% confidence interval. Prior to inferential testing, the normality of the intra-subject difference scores (pre−post) was assessed using the Shapiro–Wilk test, with *p* < 0.05 set as the significance threshold, as recommended for small sample sizes. All difference scores satisfied the normality assumption (*p* > 0.05); parametric paired-samples *t*-tests were therefore applied. The dependent variables comprised segmental joint angles at the catch and finish positions of the rowing stroke cycle, specifically trunk inclination, shoulder flexion, elbow flexion, and knee flexion, as well as positional height variables including head height, shoulder height, and hand height, and the position of the center of gravity along the X and Y axes. Outcomes were organized *a priori* into three families (segmental joint angles, body-segment heights, and center-of-gravity position). Within this structure, the Benjamini–Hochberg procedure was applied to control the false discovery rate across the 18 comparisons, and both unadjusted and adjusted *p*-values are reported. Given the modest sample and the number of comparisons, the joint-angle analyses were regarded as exploratory, and their interpretation was based on the combination of effect sizes, 95% confidence intervals, and adjusted significance rather than on unadjusted *p*-values alone. The level of statistical significance was set at *p* < 0.05 for the FDR-adjusted comparisons. Effect sizes were calculated using Cohen's *d* for paired samples, defined as the mean difference divided by the standard deviation of the differences, and interpreted as small (*d* ≥ 0.2), medium (*d* ≥ 0.5), or large (*d* ≥ 0.8) (22).

**Table 1:** Pre- and post-fatigue kinematic variables at the catch and finish phases.

Variable	Catch							Finish						
	*n*	Pre-fatigue Mean (SD)	Post-fatigue Mean (SD)	Mean difference [95% CI]	*p*	*p* (FDR)	*d*	*n*	Pre-fatigue Mean (SD)	Post-fatigue Mean (SD)	Mean difference [95% CI]	*p*	*p* (FDR)	*d*
Angulations														
Trunk (°)	15	36.6 (6.0)	39.2 (6.0)	+2.6 [−0.1, 5.3]	0.061	0.091	+0.53	15	−59.2 (7.0)	−58.5 (6.9)	+0.7 [−2.4, 3.7]	0.640	0.720	+0.12
Shoulder (°)	17	92.8 (7.8)	96.8 (8.8)	+4.0 [0.1, 7.8]	0.044*	0.072	+0.53	17	−36.6 (10.9)	−37.0 (10.2)	−0.5 [−5.7, 4.7]	0.848	0.848	−0.05
Elbow (°)	17	37.0 (8.9)	39.6 (8.9)	+2.6 [−0.3, 5.6]	0.080	0.102	+0.45	17	136.4 (5.6)	140.5 (6.4)	+4.1 [0.4, 7.9]	0.032*	0.064	+0.57
Knee (°)	13	61.8 (5.9)	57.3 (8.7)	−4.5 [−8.8, −0.3]	0.038*	0.068	−0.65	16	16.3 (6.3)	13.9 (4.2)	−2.4 [−4.5, −0.3]	0.030*	0.064	−0.60
Heights														
Head (mm)	17	1,182.0 (35.7)	1,163.8 (40.8)	−18.3 [−26.2, −10.4]	<0.001^†^	0.002*	−1.19	17	1,073.7 (68.3)	1,049.0 (65.4)	−24.7 [−40.9, −8.4]	0.005*	0.024*	−0.78
Shoulder (mm)	17	914.3 (47.4)	882.9 (36.7)	−31.4 [−45.4, −17.3]	<0.001^†^	0.002*	−1.15	17	780.3 (52.7)	765.1 (51.2)	−15.2 [−31.9, 1.5]	0.072	0.099	−0.47
Hand (mm)	16	641.1 (74.2)	626.9 (71.4)	−14.2 [−24.8, −3.7]	0.012*	0.042*	−0.72	17	989.3 (48.2)	962.8 (47.4)	−26.6 [−43.8, −9.3]	0.005*	0.024*	−0.79
Center of Gravity														
CoG X (mm)	17	−658.4 (163.6)	−701.3 (133.2)	−42.9 [−149.7, 63.9]	0.407	0.488	−0.21	17	−1,034.4 (136.0)	−1,052.4 (129.4)	−18.0 [−110.2, 74.2]	0.684	0.724	−0.10
CoG Y (mm)	17	659.4 (18.9)	647.9 (17.2)	−11.5 [−20.6, −2.3]	0.017*	0.045*	−0.64	17	565.2 (26.6)	551.5 (25.9)	−13.7 [−24.6, −2.8]	0.017*	0.045*	−0.64

Complete-case analysis; the analyzed *n* varies across variables owing to marker unavailability in some participants during one or both conditions. Values are mean (SD). Mean difference = post-fatigue−pre-fatigue, with its 95% confidence interval (CI). *d* = Cohen's *d* for paired samples (mean difference divided by the SD of the differences). *p* (FDR) = Benjamini–Hochberg false-discovery-rate-adjusted *p*-value across the 18 comparisons; this adjusted column is the reference for statistical significance. *p < 0.05; ^†^*p* < 0.001. CoG X values are anteroposterior positions relative to the laboratory coordinate origin; negative values indicate positions posterior to that origin. CoG, Center of gravity.

## 3 Results

The kinematic variables recorded at the catch and finish positions, both pre- and post-fatigue, are presented in Table 1. Fatigue induced phase-specific kinematic changes across the rowing stroke. After correction for multiple comparisons (Benjamini–Hochberg false discovery rate), statistically significant post-fatigue changes were confined to body segment heights and to the vertical position of the center of gravity, whereas the changes observed in segmental angulations did not survive correction and are therefore reported as exploratory.

At the catch position, shoulder flexion showed an unadjusted increase following the fatigue protocol (pre: 92.8 ± 7.8°; post: 96.8 ± 8.8°; *p* = 0.044; *d* =  + 0.53; 95% CI of the difference [0.1, 7.8]), which did not remain significant after FDR correction (adjusted *p* = 0.072). Knee flexion showed an unadjusted decrease (pre: 61.8 ± 5.9°; post: 57.3 ± 8.7°; uncorrected *p* = 0.038; *d* = −0.65; 95% CI [−8.8, −0.3]) that likewise did not survive correction (adjusted *p* = 0.068), suggesting a possible reduction in the degree of knee compression adopted at the catch. Trunk inclination (pre: 36.6 ± 6.0°; post: 39.2 ± 6.0°; *p* = 0.061; *d* =  + 0.53) and elbow flexion (pre: 37.0 ± 8.9°; post: 39.6 ± 8.9°; *p* = 0.080; *d* =  + 0.45) showed non-significant increases post-fatigue. At the finish position, elbow flexion showed an unadjusted increase (pre: 136.4 ± 5.6°; post: 140.5 ± 6.4°; uncorrected *p* = 0.032; *d* =  + 0.57; 95% CI [0.4, 7.9]), and knee flexion an unadjusted decrease (pre: 16.3 ± 6.3°; post: 13.9 ± 4.2°; uncorrected *p* = 0.030; *d* = −0.60; 95% CI [−4.5, −0.3]); neither remained significant after FDR correction (adjusted *p* = 0.064 for both). No changes were observed for trunk inclination (*p* = 0.640; *d* =  + 0.12) or shoulder flexion (*p* = 0.848; *d* = −0.05) at this phase.

Significant reductions in body segment heights were observed at the catch following fatigue, and these remained significant after FDR correction. Head height decreased from 1,182.0 ± 35.7 mm pre-fatigue to 1,163.8 ± 40.8 mm post-fatigue (adjusted *p* = 0.002; *d* = −1.19; 95% CI [−26.2, −10.4]), shoulder height from 914.3 ± 47.4 mm to 882.9 ± 36.7 mm (adjusted *p* = 0.002; *d* = −1.15; 95% CI [−45.4, −17.3]), and hand height from 641.1 ± 74.2 mm to 626.9 ± 71.4 mm (adjusted *p* = 0.042; *d* = −0.72; 95% CI [−24.8, −3.7]). At the finish, head height (pre: 1,073.7 ± 68.3 mm; post: 1,049.0 ± 65.4 mm; adjusted *p* = 0.024; *d* = −0.78; 95% CI [−40.9, −8.4]) and hand height (pre: 989.3 ± 48.2 mm; post: 962.8 ± 47.4 mm; adjusted *p* = 0.024; *d* = −0.79; 95% CI [−43.8, −9.3]) were significantly lower following fatigue. Shoulder height at the finish showed a reduction that did not reach significance (pre: 780.3 ± 52.7 mm; post: 765.1 ± 51.2 mm; *p* = 0.072; *d* = −0.47).

With regard to the center of gravity position at the catch, no change was detected in the anteroposterior axis (CoG X; pre: −658.4 ± 163.6 mm; post: −701.3 ± 133.2 mm; *p* = 0.407; *d* = −0.21), whereas a significant decrease that survived FDR correction was observed in the vertical axis (CoG Y; pre: 659.4 ± 18.9 mm; post: 647.9 ± 17.2 mm; adjusted *p* = 0.045; *d* = −0.64; 95% CI [−20.6, −2.3]). The same pattern was replicated at the finish: No changes were found in CoG X (pre: −1,034.4 ± 136.0 mm; post: −1,052.4 ± 129.4 mm; *p* = 0.684; *d* = −0.10), while CoG Y showed a significant post-fatigue reduction that survived correction (pre: 565.2 ± 26.6 mm; post: 551.5 ± 25.9 mm; adjusted *p* = 0.045; *d* = −0.64; 95% CI [−24.6, −2.8]).

Although group-level analyses revealed consistent directional changes for the robust outcomes, the individual response data displayed in Figures 1–3 indicate considerable interindividual variability, with some participants showing minimal or reversed responses to fatigue, particularly for joint angulations and anteroposterior CoG displacement. This variability is consistent with the exploratory status of the joint-angle findings.

**Figure 1:**
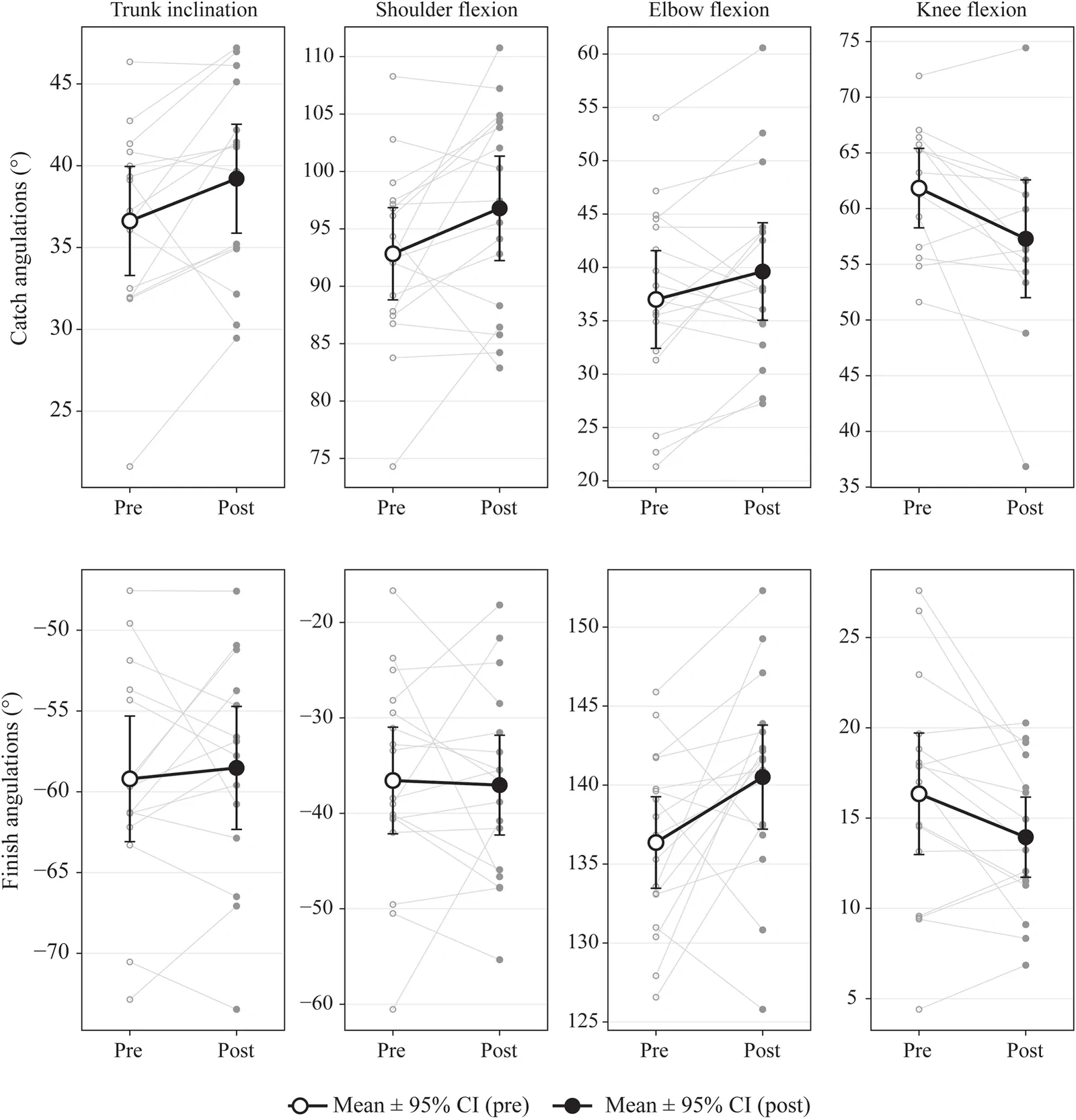
Joint angulations at the catch and finish phases before and after the fatigue protocol. Individual participant data (grey lines) with mean ± 95% CI (circles) for trunk inclination, shoulder flexion, elbow flexion, and knee flexion. Open circles = pre-fatigue; filled circles = post-fatigue.

**Figure 2:**
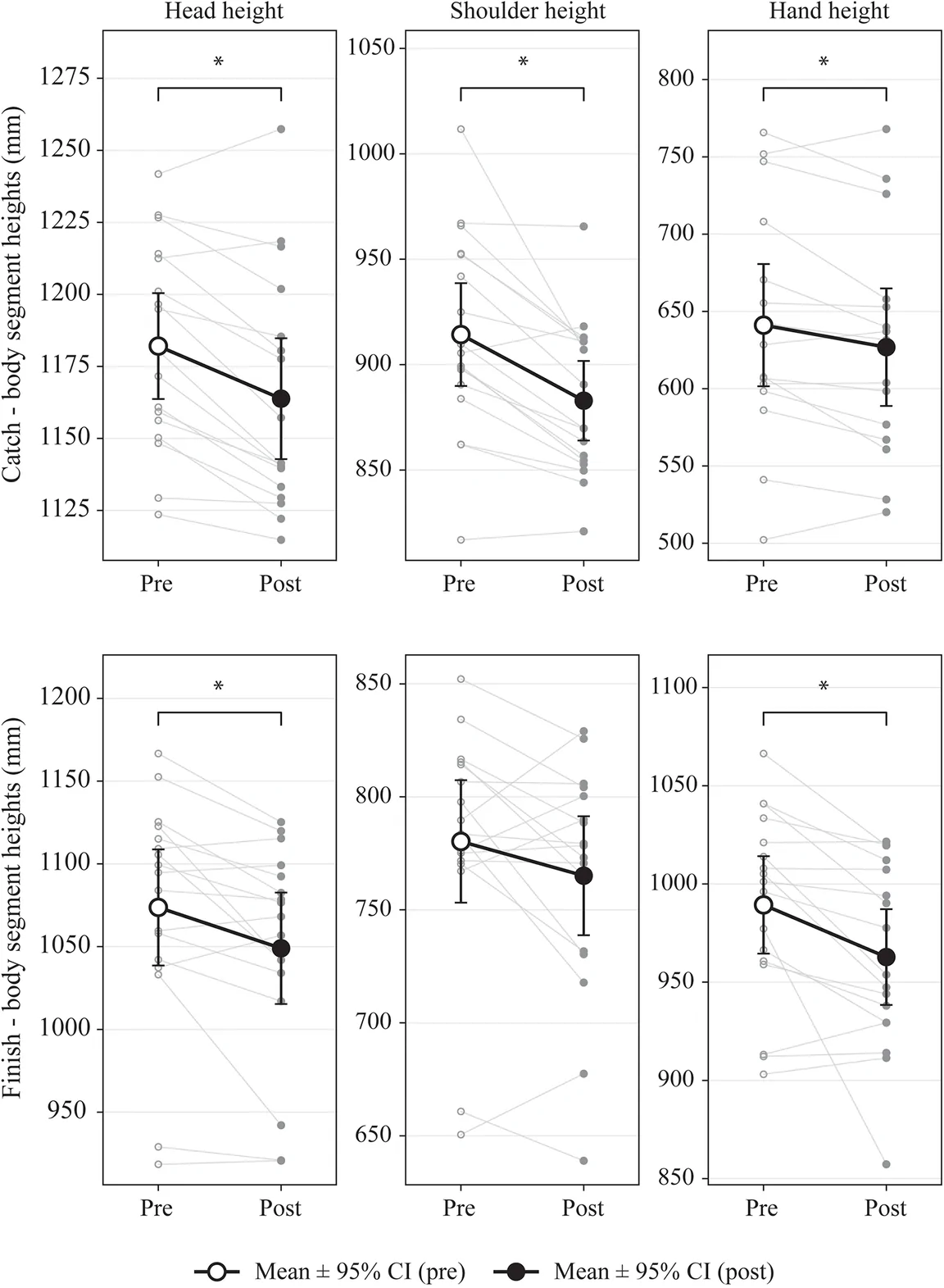
Body segment heights at the catch and finish phases before and after the fatigue protocol. Individual participant data (grey lines) with mean ± 95% CI (circles) for head, shoulder, and hand heights. Open circles = pre-fatigue; filled circles = post-fatigue. *p(*FDR*) < 0.05.

**Figure 3:**
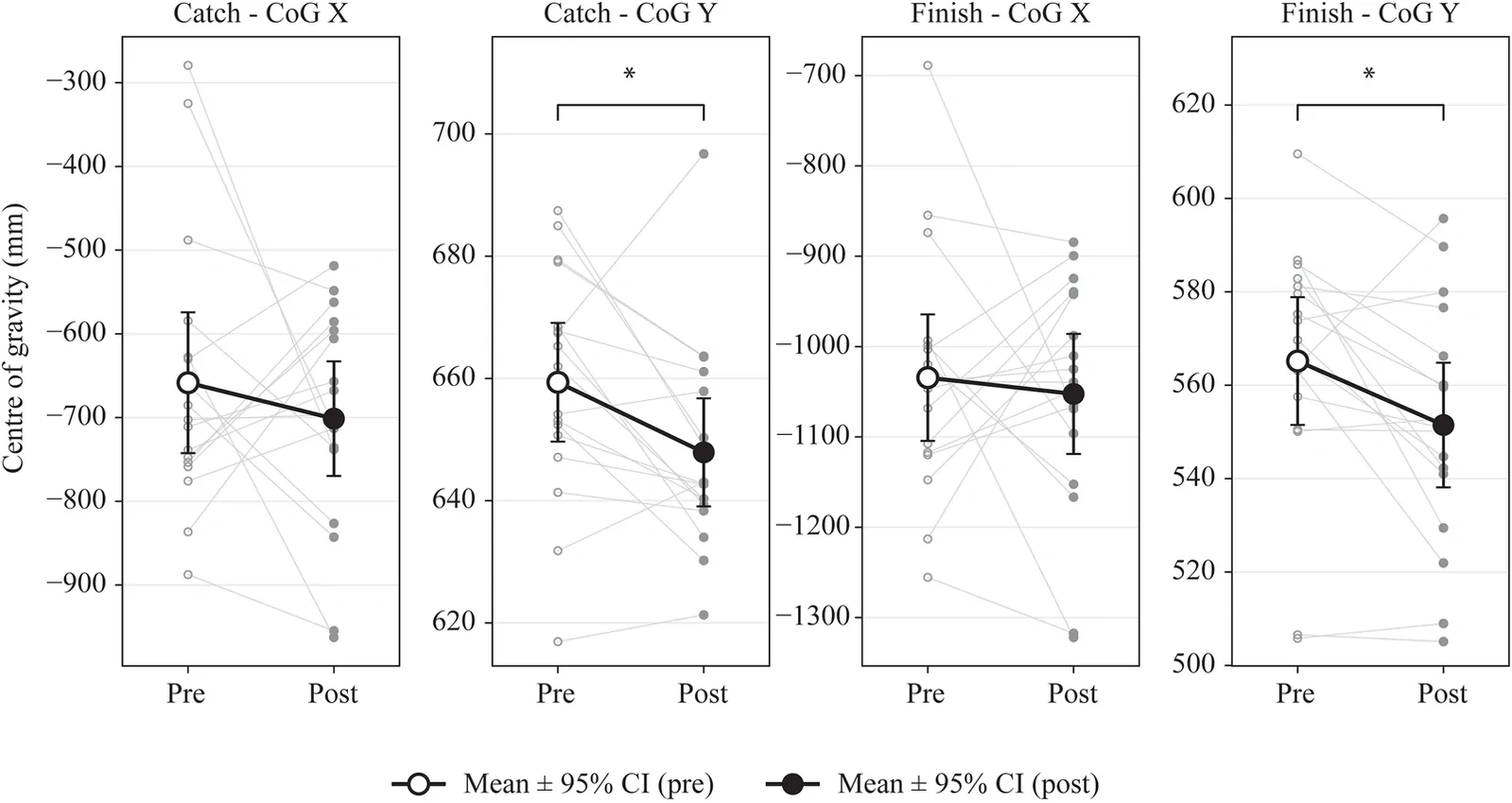
Center of gravity position at the catch and finish phases before and after the fatigue protocol. Individual participant data (grey lines) with mean ± 95% CI (circles) for anteroposterior (CoG X) and vertical (CoG Y) axes. Open circles = pre-fatigue; filled circles = post-fatigue. *p(*FDR*) < 0.05.

## 4 Discussion

This study is the first to examine how fatigue reshapes the whole-body kinematics of the rowing stroke in Traditional fixed-seat rowing, measuring joint angles, segment heights, and center of gravity (CoG) position at the catch and finish. Fatigue altered the stroke in a phase-dependent way. At the catch, shoulder flexion increased and knee flexion decreased, pointing to a less compressed entry posture; at the finish, elbow flexion increased and the knee extended further. These angular changes did not survive correction for multiple comparisons and are therefore interpreted as exploratory. The head, shoulders, and hands all sat lower after fatigue at the catch, and the head and hands at the finish, and the CoG dropped vertically without shifting along the anteroposterior axis. Overall, fatigue produced a loss of postural height, in contrast to the seat-related timing shifts that mark fatigue in Olympic rowing. Although this pattern is compatible with a trunk-related contribution, that mechanism remains hypothetical because trunk function was not measured directly and trunk inclination did not change significantly.

At the catch, the rise in shoulder flexion alongside reduced knee flexion points to a reach that was redistributed from the legs to the arms. With the pelvis fixed, entry length depends on how far the knees can be drawn up and the arms extended forward, not on seat travel (3). A smaller knee angle shortens the leg-side reach, and the rowers seem to have recovered stroke length by reaching further with the shoulder. Fatigue of the knee extensors offers a straightforward explanation: Husmann et al. found that maximal voluntary knee-extensor torque fell by 18 to 20% after a 2,000-m effort, driven mainly by reduced central activation (21). A weakened ability to hold the deeply flexed catch would limit knee flexion at entry, as we observed. In Olympic rowing, by contrast, knee flexion at the catch stays stable across a 2,000-m simulation (14); its reduction here likely reflects the heavier relative load carried by the semi-flexed leg when it works as an isometric base rather than a dynamic press (27).

The finish showed greater elbow flexion and a more extended knee, consistent with rowers leaning more on the arm draw as leg drive faded. Taken with the catch, total knee excursion across the drive shrank slightly under fatigue. Because Traditional rowing already loads roughly 60% of propulsive demand onto the trunk and arms (5, 6), any loss of leg drive has to be met further down the chain if handle travel is to hold. Olympic rowing shows a similar distal shift, with the arm pull starting progressively later as the legs, trunk, and arms fall out of sequence under load (14). Trunk inclination and shoulder flexion did not change at the finish, suggesting the rowers still reached their usual end-range lean and absorbed the extra demand through the elbow. This fits the shoulder's role as one of the largest joint excursions of the stroke and its growing importance late in the drive (12).

The most consistent change was the reduction in body-segment heights, significant for all three landmarks at the catch and for head and hand heights at the finish (shoulder height at the finish showed a non-significant reduction). This pattern may reflect a reduced capacity of the trunk extensors to maintain extension, expressed as a whole-body loss of postural height. Spinal flexion is well known to increase as rowing effort accumulates, whether during long low-intensity sessions (13, 32) or race-pace efforts (14, 33), and the drift is sharpest on the ergometer, where some rowers exceed their standing flexion limit (29). The height reductions here are the external counterpart of that flexion: as posture gives way, the upper-body landmarks are carried down with it. This would be consistent with fatigue of the dorsal musculature. Because trunk-extensor function was not measured directly (no EMG or force data were recorded), the following interpretation is proposed as one plausible mechanism rather than a demonstrated one. Willwacher et al. reported increased thoracic curvature in all-out ergometer rowing alongside falling mean frequencies in the latissimus dorsi and descending trapezius, the muscles that stabilize the thorax (34). Consistent with this, a maximal 2,000-m row has recently been shown to acutely increase the tone and stiffness of the latissimus dorsi and other prime movers in rowers (35). Central and strength factors compound the effect: corticospinal drive to the erector spinae stays depressed after rowing (36), and elite rowers already carry low extensor-to-flexor strength ratios (37). Since the fixed seat loads the trunk more heavily than the sliding seat (10), and the trunk is the principal contributor to the fixed-seat stroke, it is plausible that the trunk extensors are among the first segments to fatigue, which would be consistent with the postural changes observed here.

The CoG data tells the same story. Its vertical position fell at both the catch and the finish, while its anteroposterior position did not move. Fatigue lowered the rowers; it did not push them forward or back. With a fixed pelvis there is little room to redistribute mass fore and aft, so the most evident change is a descent of the upper-body landmarks. Because trunk inclination did not change significantly and spinal flexion was not measured, this descent should not be interpreted as direct evidence of trunk flexion. Olympic rowing instead tends to express fatigue through seat timing and drive sequencing (14). The consistent vertical lowering of the CoG seen here may be a fatigue signature specific to fixed-seat rowing, and its appearance at both phases argues that it is a real adaptation rather than noise.

These results can be read against only earlier study of fatigue and whole-body coordination in Traditional rowing. Alano et al. found no significant change in spatiotemporal coordination after a fatiguing bout, though with clear trends and wide inter-individual variation (16). Method differences may explain why our effects were sharper. We used an incremental test to volitional exhaustion with an objective failure criterion rather than a Borg-scale bout; we measured discrete angles, heights, and CoG at the catch and finish rather than continuous coordination indices. Moreover, the fixed seat directs mechanical fatigue predominantly onto the trunk and upper body. This localized demand may make technical adjustments easier to detect than in the sliding-seat configuration, which Alano et al. described as minimally sensitive to fatigue. That changes appeared across angles, heights, and CoG together suggests segment-level measures may track fatigue-related technique loss more sensitively than global coordination metrics, at least in fixed-seat rowing.

The findings also matter clinically. Rowing carries one of the highest low-back-pain burdens in sport, with lifetime prevalence reaching 94% (38), and repeated end-range spinal flexion under cyclical load is an established injury risk (18, 29). Falling segment heights and a sinking CoG provide a simple, whole-body readout of fatigue-related postural change, and thus a potential way to flag when technique begins to break down. As trunk flexion was not measured directly and trunk inclination did not change significantly, these changes should be regarded as descriptive postural markers rather than direct indicators of trunk flexion. Practically, this favors conditioning that builds fatigue resistance in the trunk extensors and posterior chain, and coaching cues that keep the trunk tall as fatigue sets in. In other sports, targeted interventions have been shown to attenuate fatigue-related decrements in performance and flexibility (39), supporting the rationale for conditioning strategies aimed at building fatigue resistance in rowers. Injury outcomes were not assessed in this study; the clinical implications discussed here are therefore hypothetical and require prospective validation against injury data. Multisegmental monitoring of the kind used here could help coaches detect early fatigue-related technique loss. Because prior work links such postural changes to increased spinal loading, this approach may also have future relevance for injury-prevention strategies, although this remains to be tested against injury outcomes.

Some limitations temper these conclusions. First, the number of comparisons relative to the sample size increases the risk of Type I error; although a false-discovery-rate correction was applied within prespecified families, the joint-angle changes did not survive adjustment and are therefore treated as exploratory, whereas the segment-height and center-of-gravity findings withstood correction and provide a more robust basis for inference. Second, kinematics were restricted to the sagittal plane, and although heart rate and perceived exertion confirmed high systemic fatigue, trunk-extensor function was not measured directly (no EMG); the proposed muscular mechanisms therefore remain inferential. Some markers detached during the effort and were repositioned onto skin landmarks marked before the session to minimize positional error; unreliable variables were excluded per participant, producing the variable sample size across outcomes. The number of repositioning events was not systematically recorded, and the repositioning error, the session-specific calibration error, and the test–retest reliability of the extracted variables were not quantified, which is acknowledged as a limitation. In addition, the segmental inertia model used by the 3DMA software for the center-of-gravity estimate was not independently specified, so cross-study comparisons of CoG values should be made with caution. Third, the target intensity (70% of estimated VO₂max power) was derived from training records rather than measured on the day, and the instantaneous cadence and power of the recordings were not logged; although the prescribed target was identical across conditions and supervised in real time, a difference in actual intensity between the pre- and post-fatigue recordings cannot be excluded and may have contributed to the observed kinematic changes. Testing was conducted indoors on a fixed-seat ergometer rather than on water, ensuring reproducibility but removing the on-water instability that may accentuate fatigue effects (40); the post-fatigue recording followed a short passive rest interval of 5 min, during which some recovery may have occurred. Finally, the sample was elite and male, which limits generalizability: documented sex differences in trunk and pelvic kinematics (12, 41), caution against extending the findings to women; the highly consolidated technique of these ACT-division athletes may not represent recreational or sub-elite rowers, who fatigue earlier and less stably; and the mechanical distinctiveness of the fixed seat, which redistributes load onto the trunk and upper limbs, means these results should not be extrapolated to Olympic sliding-seat rowing. Future studies should move this work onto the water, record EMG and physiological fatigue markers during the stroke, individualize intensity, and include female and sub-elite rowers. Because fatigue responses vary so widely between individuals (16), profiling who adapts and how may prove the most useful step for targeting injury prevention in this under-studied modality.

## 5 Conclusions

Fatigue altered joint angles, segment heights, and center of gravity position differently at the catch and finish phases. At the catch, shoulder flexion increased and knee flexion decreased, pointing to a less compressed entry posture; at the finish, elbow flexion rose while knee flexion fell, consistent with a modified arm pull and greater leg extension.

The concurrent lowering of head and hand heights, and of shoulder height at the catch, reflects a systematic reduction in postural height under sustained rowing.

The vertical drop in center of gravity, with no corresponding shift in the anteroposterior axis, suggests that fatigue compromised postural height rather than redistributing body mass along the stroke. These findings support the use of multisegmental kinematic monitoring as a practical tool for fatigue-related technique assessment in Traditional rowing.

## Data Availability

The raw data supporting the conclusions of this article will be made available by the authors, without undue reservation.
